# HIV viral load suppression rates among adults and children living with HIV in the North West Region of Cameroon: A call for action!

**DOI:** 10.1371/journal.pone.0316399

**Published:** 2025-01-31

**Authors:** Gladys Fosah E. Tayong, Melissa Sander, Comfort Vuchas, Moses Samje, Vera Kum, Pascal Enokbonong, Eugene Yeika, Ndanga Bekombo, Paul Nyibio, Vanessa B. Ngwani, Justin Ndié, Njamnshi wepnyu Yembe, Gloria Ashuntangtang

**Affiliations:** 1 Faculty of Health Sciences, University of Bamenda, Bambili, Cameroon; 2 Regional Technical Group for the HIV Response, North West Region, Bamenda, Cameroon; 3 Tuberculosis Reference Laboratory, Bamenda, Cameroon; 4 Faculty of Economics and Management Sciences, University of Bamenda, Bambili, Cameroon; 5 ICAP, Garoua, Cameroon; 6 Kumbo East Health District, Regional Delegation of Public Health North West Region, Kumbo, Cameroon; 7 Division of Operational Research in Health, Ministry of Public Health, Yaounde, Cameroon; United States Agency for International Development (USAID), NIGERIA

## Abstract

**Introduction:**

HIV continues to be a major public health problem in Cameroon where 2.7% of the population aged 15 to 49 are living with HIV. The prevalence remains higher in females, 3.4% versus 1.9% in males. The North West region of Cameroon has a higher prevalence than the national average; 4% in the general population, 5.8% in females and 1.6% in males. Despite the availability of pediatric HIV treatment, gaps in outcomes of children living with HIV (CLHIV) persists. This study aimed to compare the HIV viral suppression rates among adults and children living with HIV receiving antiretroviral therapy (ART) in the North West Region of Cameroon. In this study we hypothesized that the virologic outcome of children living with HIV is unfavorable compared to adults.

**Methodology:**

This study was a comparative cross-sectional analytical study with secondary analysis of Viral load laboratory database. Data was collected in June 2023. We systematically collected deidentified data on viral load test of all PLHIV with HIV viral load results in the Bamenda TB reference laboratory database for the period January 1, 2022 to December 31, 2022. Data was analyzed using Stata SE 14.2. Categorical variables were described using absolute and relative frequencies. These were compared using Pearson’s chi-squared test. Quantitative variables were described using mean as a characteristic of central tendency and standard deviation (SD) as a characteristic of dispersion.

**Results:**

Analysis revealed that of the 23,814 PLHIV whose viral load test was done at the Bamenda TB reference laboratory during the year 2022, 17,364(73%) were female and 1436 (6%) were children. The level of HIV viral suppression (viral load<1000 copies/ml) in children was lower compared to that of adults (80% vs. 95%; p<0.001). Females had lower viral suppression than males (94% vs. 91%; p<0.001).

**Conclusion:**

The results revealed a significant lower HIV viral suppression rate in children than in adults. Women also had a significantly higher HIV viral suppression than males. Findings indicated that HIV viral suppression rate is significantly higher for routine viral load testing than for targeted testing after enhanced adherence counselling, thus the need to systematically offer enhanced adherence counselling and other evidence-based support interventions in routine care to all PLHIV to avoid persistent high viral non-suppression. Action is needed to tackle all known factors contributing to high viral non-suppression in children living with HIV and to help achieve 95% viral suppression rate in CLHIV in the North West region of Cameron.

## 1. Introduction

Worldwide, gross disparities in HIV service delivery and outcomes exist between adults and children living with HIV [1–4]. The UNAIDS targets of 95-95-95 requires that for all age groups and subpopulation type, 95% of People living with HIV(PLHIV) should know their HIV status; 95% of PLHIV should be placed on antiretroviral therapy and 95% of PLHIV on ART should have viral suppression by 2030 [5]. These targets, especially the third 95 have not been achieved for Children [2]. According to WHO, of the 39 million people living with HIV worldwide, 1.5 million are children aged 0–14 years [2]. West and central Africa is responsible for 26% of children living with HIV Globally [6]. Despite the fact that 91% of children 0–14 years with known HIV positive status in the world were placed on treatment, only 81% of children aged 0–14 years who were receiving antiretroviral therapy had suppressed viral load compared to 93% among all PLHIV [2]. UNAIDS reports that achieving viral suppression in children will lead to reduced new infections, reduced AIDS related deaths and increased investments in HIV [5]. Unfortunately, several risk factors of non-suppression like late ART initiation, the type of ARV regimen, poor adherence to medication, advanced HIV disease, history of tuberculosis, structural challenges and low socioeconomic status, lack of support to caregivers, health system factors constitute persistent roadblocks [3, 4, 7–12].

These disparities in HIV services and treatment outcomes have also been reported in Cameroon. Cameroon being the second highest HIV hit country in west and central Africa has an HIV prevalence of 2.7% in the population aged 15 to 49 and harbors about 480,000 PLHIV of the 39 million people estimated to be living with HIV in the world [2, 13, 14]. The viral suppression rate in children remains low in all 10 regions of Cameroon [13]. The North West region of Cameroon has a higher HIV prevalence of 4% with a disproportionate prevalence of 5.8% in females and 1.6% in males. Even though the disparities in HIV services and viral load suppression rates among children is evident in all subnational entities, the situation is aggravated in the North West and South West regions of Cameroon experiencing protracted civil unrest and armed conflict. Protracted emergencies have been described to be a risk factor of poor outcomes for children living with HIV [3].

The HIV treatment guidelines in Cameroon, in line with the 2021 WHO guidelines recommend, optimized ART regimens for both adults and Children [15]. With the introduction of optimized regimens and Dolutegravir-containing regimens in 2019 in the general population and eventually to children in 2021, there has been an increase in viral load suppression rates in all population groups [13, 16]. Other factors that have contributed to increased viral suppression among PLHIV include government initiatives like the “no user fees in HIV services”; the implementation of pediatric training centers of excellence; good availability of optimized pediatric HIV treatment regimens; an increase in the number of viral load machines and the contribution of clinical and community implementing partners. Despite all these, viral load coverage and suppression in children remains low, retarding progress toward the 95% viral suppression rates in Children. Monitoring treatment in CLHIV will reveal the virologic outcome or status of these children and help in the design of interventions to improve the continuum of care for Children living with HIV [17]. In an effort to draw more attention to the virologic outcome of Children living with HIV in the North West region of Cameroon, we conducted this comparative cross-sectional analytical study using the HIV viral load testing data of the TB reference Laboratory. This study aimed to compare the HIV viral load suppression rate among adults and children living with HIV on ART in the North West region of Cameroon. In our study we hypothesized that the HIV viral load suppression rate in children is less than in adults. This study is not to undermine the progress made in HIV viral suppression rate in children since 2016 but to call for evidence-based interventions to improve the outcomes of children living with HIV in the North West Region and in Cameroon at large.

## 2. Methodology

### 2.1. Type of study

This was a comparative cross-sectional analytical study with secondary analysis of Viral load laboratory database.

### 2.2. Study location

The study took place in Bamenda at the Tuberculosis reference laboratory (TBRL). The TB reference laboratory is a state recognised laboratory responsible for Viral load testing, early infant diagnosis of HIV and Tuberculosis testing in the North West Region of Cameroon. The North West Region is one of the 10 administrative units of Cameroon. There are about 420 health facilities and 387(92%) of these health facilities offer antiretroviral therapy to over 41,000 PLHIV in the region. These health facilities collect whole blood from PLHIV, centrifuge and transport the plasma to the TBRL which is the Ministry of Public Health approved laboratory for conventional viral load testing. Here the PCR test is done by extraction, amplification and quantification. Three categories of results given by the laboratory include: undetectable viral load <50 copies/ml; detectable below 1000 copies/ml which is low level viraemia and 1000 copies/ml and above which is considered unsuppressed viral load.

### 2.3. Duration of the study

Data for this study was collected in June 2023. Analysis, write up of initial draft manuscript, final manuscript including review by all authors took place from July 2023 to May 2024.

### 2.4. Study population and inclusion criteria

Included in this study were viral load results of children and adults living with HIV receiving ART in the North West Region tested for viral load whose results were recorded in the laboratory database between 1^st^ January 2022 to 31^st^ December 2022.

### 2.5. Technique for recruiting or selecting individuals and Sample size

All HIV viral load test results in the Bamenda Tuberculosis Reference Laboratory database between January 1, 2022 and December 31, 2022 were systematically included in the study. The initial sample size was 23,814.

### 2.6. Data collection tool and data processing

The data for this study was anonymized and extracted from the laboratory database for viral load testing to an excel sheet. The deidentified data was cleaned. The variables of interest were identified and data completeness was accessed for each variable. All variables considered in the study had very little missing data, namely age (2.8%), Sex (0.43%), duration on ART (4.58%), reason for viral load testing (0%) and viral load test results (0%).

### 2.7. Data analysis

Data were anonymized before access and analysis. The variables of interest were age, sex, duration on ART, reason for viral load testing and viral load result. Age was coded into 2 age groups 0–18 for children and above 18 years for adults. Sex was categorized as either male or female; the Viral load status was categorized as suppressed for viral load results less than 1000 copies/ml and unsuppressed for Viral load 1000 copies/ml and above. Data was analyzed using Stata SE 14.2. Categorical variables were described using absolute and relative frequencies. These were compared using Pearson’s chi-squared test. The quantitative variables were described using the mean as a characteristic of central tendency and the standard deviation (SD) as a characteristic of dispersion. Comparisons of these variables were made using the t-test. Multiple logistic regression was used to examine associations between viral suppression and the variables sex, age group, duration on ART and reason for viral load testing. The significance level considered for all hypothesis tests was 5%.

### 2.8. Ethical considerations

The study protocol was submitted to the Regional Ethics Committee for Human Health Research in the Northwest Region of Cameroon for review and ethical clearance. Ethical clearance No 2023/17/CERSH-NW was obtained in May 2023 prior to data collection and analysis. Due to the fact that data was anonymized, informed consent was waived. The head of the TB reference Laboratory also signed a letter of collaboration and authorized data collection.

## 3. Results

Analysis on Table 1 revealed that of the 23,814 PLHIV whose viral load test was done at Bamenda TB reference laboratory during the year 2022, 17,364(73%) were women and 1,436 (6%) children. The average duration on ART was 9.1 (SD = 5.9) years. Routine viral load tests were predominant, 95% of the total. The average time between sample collection from patients and receipt by the laboratory was 5.3 (SD = 6.9) days. Samples spent on an average 17.4 (SD = 25.6) days in the laboratory before the viral load test was performed. The viral suppression rate in our study population was 93.5%. This indicator in children was lower compared to that of adults (80% vs. 95%; p<0.001). Women had better viral suppression rate than men (94% vs. 91%; p<0.001).

**Table 1:** HIV viral load results of PLHIV on ART according to certain factors, North West Region, Cameroon, 2022.

Variable	All	Viral Load result		P-value
		Unsuppressed	Suppressed	
	n (%)	n (%)	n (%)	
**Sex**				<0.001
Female	17364 (72.9%)	982 (5.7%)	16382 (94.3%)	
Male	6347 (26.7%)	549 (8.6%)	5798 (91.4%)	
Missing	103 (0.4%)			
**Age group**				<0.001
Children	1436 (6.0%)	287 (20.0%)	1,149 (80.0%)	
Adults	21711 (91.2%)	1201 (5.5%)	20510 (94.5%)	
Missing	667 (2.8%)			
**Viral load indication**				<0.001
Routine	22645 (95.1%)	1190 (5.3%)	21455 (94.7%)	
Repeat after enhanced adherence counselling	968 (4.1%)	296 (30.6%)	672 (69.4%)	
Targeted Clinical/Immunological Failure	118 (0.5%)	41 (34.7%)	77 (65.3%)	
Others	83 (0.3%)	12 (14.5%)	71 (85.5%)	
**Duration on ART in years**, mean (SD)	9.1 (5.9) (n = 22761)	8.1 (5.8)	9.2 (5.9)	<0.001
**Time between sample collection and receipt at the laboratory in days**, mean (SD)	5.3 (6.9) (n = 23473)	6.2 (7.4)	5.2 (6.8)	<0.001
**Time between receipt of sample by laboratory and viral load test in days**, mean (SD)	17.4 (25.6) (n = 23807)	14.7 (22.2)	17.5 (25.8)	<0.001

According to Table 2, among children there is no significant difference in suppression rates between females and males (80.8% vs. 79.2%; p = 0.44). Among 968 PLHIV for whom viral load test was done after enhanced adherence counselling, more than 69% had a suppressed viral load compared to 57.8% in children.

**Table 2:** HIV viral load results of children on ART according to certain factors, North West Region, Cameroon, 2022.

Variable	All	Viral Load result		P-value
		Unsuppressed	Suppressed	
	n (%)	n (%)	n (%)	
**Sex**				0.44
Female	778 (54.2%)	149 (19.2%)	629 (80.8%)	
Male	649 (45.2%)	135 (20.8%)	514 (79.2%)	
Missing	9 (0.6%)			
**Viral load indication**				<0.001
Routine	1242 (86.5%)	201 (16.2%)	1041 (83.8%)	
Repeat after enhanced adherence counselling	180 (12.5%)	76 (42.2%)	104 (57.8%)	
Targeted Clinical/Immunological Failure	8 (0.6%)	6 (75.0%)	2 (25.0%)	
Other	6 (0.4%)	4 (66.7%)	2 (33.3%)	
**Duration on ART in years**, mean (SD)	7.9 (5.2) (n = 1398)	7.5 (4.2)	8.0 (5.4)	0.16
**Time between sample collection and receipt at the laboratory in days**, mean (SD)	5.9 (7.1) (n = 1420)	6.4 (7.6)	5.8 (7.0)	0.18
**Time between receipt of sample by laboratory and viral load test in day**, mean (SD)	14.8 (23.0) (n = 1435)	13.2 (21.4)	15.2 (23.4)	0.19

**Fig 1:**
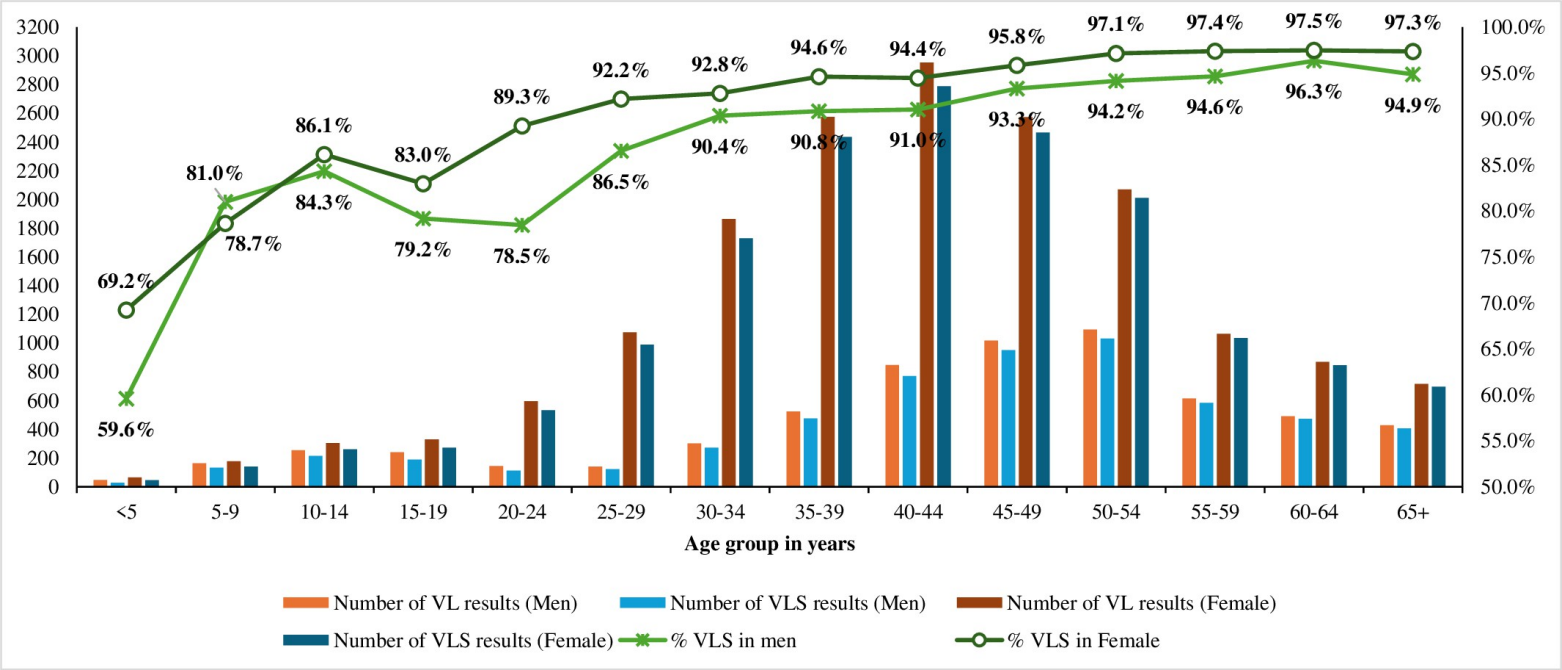
HIV Viral Load results of PLHIV on ART by sex and age, North West, Cameroon, 2022.

With the exception of age group 5–9 years, the HIV viral suppression curve for women is above that for men in all age groups (Fig 1)

Regarding the analysis of the association between HIV viral suppression rates and certain factors using multiple logistic regression, Table 3 highlights a higher odds of viral suppression in adults compared to children (Odds ratio = OR = 3.25; p<0.001). The result by gender indicates that the odds of viral suppression in females is 40% higher than in males (OR = 1.40; p<0.001). Also, an increase of one year on ART increases the odds of viral suppression by 2% (OR = 1.02; p<0.001). Finally, as opposed to viral loads carried out routinely, a low odds of viral suppression is recorded for viral load test after enhanced adherence counselling (OR = 0.15; p<0.001) and also for viral load done due to suspicion of clinical or immunological failure (OR = 0.09; p<0.001).

**Table 3:** Association between HIV viral suppression rate and the factors age, sex, duration on ART and indication of viral load test, North West, Cameroon, 2022.

Variables		Unadjusted estimate			Adjusted estimate		
		OR	p-value	95% CI	OR	p-value	95% CI
Age	**Reference: Children**	1			1		
	Adults	4.14	<0.001	3.598–4.767	3.25	<0.001	2.783–3.785
Sex	**Reference: Male**	1			1		
	Female	1.58	<0.001	1.421–1.761	1.40	<0.001	1.246–1.574
Duration on ART in years	Continuous	1.02	<0.001	1.015–1.028	1.02	<0.001	1.013–1.027
Viral Load indication	**Reference: Routine**	1			1		
	Repeat after enhanced adherence	0.14	<0.001	0.108–0.145	0.15	<0.001	0.122–0.167
	Target Clinical/Immunological Failure	0.09	<0.001	0.071–0.149	0.09	<0.001	0.062–0.140
	Other	0.36	0.001	0.189–0.644	0.37	0.003	0.185–0.711

**OR**: Odds ratio; **CI**: Confidence Interval

## 4. Discussion

Analysis revealed that of the 23,814 viral load test results at the Bamenda TB reference laboratory during the year 2022, 17,364(73%) were females and 1436 (6%) children. Globally children comprise 4% of PLHIV in 2021 [18]. In our sample children comprised 6% similar to the proportion of children among PLHIV which is 6,2% in the North west region [19]. This could imply similar uptake of viral load testing in children and adults. In our study the viral load suppression rate in children was unfavorable or low compared to adults (80% vs. 95%; p<0.001). Our null hypothesis was rejected. Even though much progress has been made in the viral suppression rates in children, they are still lagging behind. Our finding is consistent with a study by the leDEA collaboration in 16 countries that revealed that viral suppression rates in children and adult on ATRT increased over time with the children always lagging behind, with viral suppression after 3 years that changed in adults from 45% to 90% and in children from 60.6% to 80.4% [20]. Similarly, a multiregional study in 30 countries revealed the proportion of children and adolescent on ART with viral suppression was lower compared to adults at 1 year, 2 years and 3 years after ART initiation, 64% Vs 79%, 62% vs 72% and 59% vs 65% respectively [21]. Fokam et al in 2019 also reported lower viral suppression rates in adolescents on ART [22]. In a meta-analysis including 72 studies Boerma et al reported suboptimal viral suppression rates in children on ART in low and middle income countries of 62.5% (95%CI, 53.3–72.6) in the period 2010 and later [23]. Evidence on lower viral suppression rates in children cannot be ignored. In our study even though females had better viral suppression than males (94% vs. 91%; p<0.001) in the general population, progress toward achieving the UNAIDS third 95% target in the general population was more evident.

Our study reveals that among children there is no significant difference in suppression rates between females and males (80.8% vs. 79.2%; p = 0.44). The factors associated with virologic non-suppression in children are well documented and poor adherence to medication is an important factor. In our study among 968 PLHIV with previous virologic non-suppression who were reported to have been offered at least 3 consecutive monthly enhanced adherence counselling sessions with observed improvement in adherence, 64% PLHIV had a suppressed viral load as against 57.8% in children with previous virologic non-suppression. Several studies corroborate with our findings [24–30]. In our study, the odds of viral suppression was higher for routine viral load testing compared with repeated viral load testing after 3 enhanced adherence counselling sessions (1 Vs .14). Jobanputra et al in their study, reported that receiving enhanced adherence counselling was not associated with re-suppression [31]. Their study population had a smaller sample size and 16% non-suppression compared to 6.5% in our population. These findings indicate the limitations of enhanced adherence counselling after virologic failure, thus the need to systematically offer enhanced adherence counselling in routine care to all PLHIV, and not wait for non-suppression to set in.

## 5. Conclusion

Our study revealed that in the North West region of Cameroon, HIV viral suppression rate among PLHIV was 93.5% in 2022 with a lower suppression rate of 80% in children. Much progress has been made toward the third UNAIDS 95% fast tract target in PLHIV but children are still left behind. Even though males have lower suppression rates than females, the situation in children remains critical. Evidence from our studies shows that considering factors contributing to non-suppression and integrating evidence-based interventions may bring a solution to the puzzle. The lower suppression rates after enhanced adherence counselling compared to routine viral load testing, which was more marked in children, emphasizes the need to systematically offer enhanced adherence counselling in routine care to all PLHIV and not only to PLHIV with high viral load. While considering this in routine care, the effect of the protracted civil unrest and armed conflict on children living with HIV in the North West region of Cameroon should also be studied and support interventions provided. Action is needed to tackle all known factors contributing to higher viral non-suppression in children living with HIV to help achieve 95% HIV viral suppression rate in CLHIV in the North West region of Cameroon.

## Supporting information

S1 Fig HIV viral load results of PLHIV on ART by sex and age, North West, Cameroon, 2022 (details).
(TIF)

S1 File (XLSX)

## References

[1] 2022-global-aids-update_en.pdf [Internet]. [cited 2023 May 27]. Available from: https://www.unaids.org/sites/default/files/media_asset/2022-global-aids-update_en.pdf

[2] j0294-who-hiv-epi-factsheet-v7.pdf [Internet]. [cited 2024 Jan 8]. Available from: https://cdn.who.int/media/docs/default-source/hq-hiv-hepatitis-and-stis-library/j0294-who-hiv-epi-factsheet-v7.pdf

[3] Chamla D, Luo C, Idele P. Children, HIV, emergencies and Sustainable Development Goals: roadblocks ahead and possible solutions. J Int AIDS Soc. 2018 Feb 27;21(Suppl Suppl 1):e25046. doi: 10.1002/jia2.25046 29485728 PMC5978666

[4] Understanding and Improving Viral Load Suppression in Children with HIV In Eastern and Southern Africa | Children & AIDS [Internet]. [cited 2023 Jun 9]. Available from: https://www.childrenandaids.org/vls-clhiv

[5] 201506_JC2743_Understanding_FastTrack_en.pdf [Internet]. [cited 2024 Feb 21]. Available from: https://www.unaids.org/sites/default/files/media_asset/201506_JC2743_Understanding_FastTrack_en.pdf

[6] Step_Up_the_Pace_West_and_Central_Africa-ENG.pdf [Internet]. [cited 2024 May 31]. Available from: https://www.unicef.org/media/48656/file/Step_Up_the_Pace_West_and_Central_Africa-ENG.pdf

[7] Mageda K, Kulemba K, Olomi W, Kapologwe N, Katalambula L, Petrucka P. Determinants of nonsuppression of HIV viral load among children receiving antiretroviral therapy in the Simiyu region: a cross-sectional study. AIDS Res Ther. 2023 Apr 13;20(1):22. doi: 10.1186/s12981-023-00515-1 37055786 PMC10099818

[8] Belay GM, Engeda EH, Ayele AD. Late antiretroviral therapy initiation and associated factors among children on antiretroviral therapy at University of Gondar Comprehensive Specialized Hospital, Gondar, Northwest Ethiopia: a cross-sectional study. BMC Res Notes. 2019 May 7;12:255. doi: 10.1186/s13104-019-4279-z 31064418 PMC6505062

[9] Shiferaw MB, Endalamaw D, Hussien M, Agegne M, Amare D, Estifanos F, et al. Viral suppression rate among children tested for HIV viral load at the Amhara Public Health Institute, Bahir Dar, Ethiopia. BMC Infect Dis. 2019 May 14;19(1):419. doi: 10.1186/s12879-019-4058-4 31088496 PMC6518745

[10] Kakkar F, Lee T, Hawkes MT, Brophy J, Lindy S, Singer J, et al. Challenges to achieving and maintaining viral suppression among children living with HIV. AIDS. 2020 Apr 1;34(5):687. doi: 10.1097/QAD.0000000000002454 31794519

[11] Afrane AKA, Goka BQ, Renner L, Yawson AE, Alhassan Y, Owiafe SN, et al. HIV virological non-suppression and its associated factors in children on antiretroviral therapy at a major treatment centre in Southern Ghana: a cross-sectional study. BMC Infect Dis. 2021 Aug 2;21(1):731. doi: 10.1186/s12879-021-06459-z 34340689 PMC8330060

[12] Nabukeera S, Kagaayi J, Makumbi FE, Mugerwa H, Matovu JKB. Factors associated with virological non-suppression among HIV-positive children receiving antiretroviral therapy at the Joint Clinical Research Centre in Lubowa, Kampala Uganda. PLOS ONE. 2021 Jan 27;16(1):e0246140. doi: 10.1371/journal.pone.0246140 33503074 PMC7840004

[13] CNLS. Rapport Annuel 2022 de lutte contre le VIH/SIDA Au Cameroun. 2022.

[14] Institut National de la Statistique (INS) et ICF. Enquête Démographique et de Santé du Cameroun 2018. [Internet]. 2020. Available from: https://dhsprogram.com/publications/publication-FR360-DHS-Final-Reports.cfm

[15] Consolidated guidelines on HIV prevention, testing, treatment, service delivery and monitoring: recommendations for a public health approach [Internet]. [cited 2023 Jun 8]. Available from: https://www.who.int/publications-detail-redirect/9789240031593 34370423

[16] Fokam J, Nka AD, Mamgue Dzukam FY, Efakika Gabisa J, Bouba Y, Tommo Tchouaket MC, et al. Viral suppression in the era of transition to dolutegravir-based therapy in Cameroon: Children at high risk of virological failure due to the lowly transition in pediatrics. Medicine (Baltimore). 2023 May 19;102(20):e33737. doi: 10.1097/MD.0000000000033737 37335723 PMC10194733

[17] Pham MD, Nguyen H, Anderson D, Crowe S, Luchters S. Viral load monitoring for people living with HIV in the era of test and treat: progress made and challenges ahead–a systematic review. BMC Public Health. 2022;22(1):1–23. 35710413 10.1186/s12889-022-13504-2 PMC9202111

[18] 2022-global-aids-update-summary_en.pdf [Internet]. [cited 2024 Jan 6]. Available from: https://www.unaids.org/sites/default/files/media_asset/2022-global-aids-update-summary_en.pdf

[19] Tayong. Annual Report of HIV Response in the North West Region of Cameroon. RTG HIV North West Region; 2022.

[20] Jiamsakul A, Kariminia A, Althoff KN, Cesar C, Cortes CP, Davies MA, et al. HIV Viral Load Suppression in Adults and Children Receiving Antiretroviral Therapy—Results From the IeDEA Collaboration. JAIDS J Acquir Immune Defic Syndr. 2017 Nov 1;76(3):319. doi: 10.1097/QAI.0000000000001499 28708808 PMC5634924

[21] Han WM, Law MG, Egger M, Wools-Kaloustian K, Moore R, McGowan C, et al. Global estimates of viral suppression in children and adolescents and adults on antiretroviral therapy adjusted for missing viral load measurements: a multiregional, retrospective cohort study in 31 countries. Lancet HIV. 2021 Dec 1;8(12):e766–75. doi: 10.1016/S2352-3018(21)00265-4 34856180 PMC8782625

[22] Fokam J, Sosso SM, Yagai B, Billong SC, Djubgang Mbadie RE, Kamgaing Simo R, et al. Viral suppression in adults, adolescents and children receiving antiretroviral therapy in Cameroon: adolescents at high risk of virological failure in the era of “test and treat.” AIDS Res Ther. 2019 Nov 19;16(1):36. doi: 10.1186/s12981-019-0252-0 31744517 PMC6864925

[23] Boerma RS, Boender TS, Bussink AP, Calis JCJ, Bertagnolio S, Rinke de Wit TF, et al. Suboptimal Viral Suppression Rates Among HIV-Infected Children in Low- and Middle-Income Countries: A Meta-analysis. Clin Infect Dis. 2016 Dec 15;63(12):1645–54. doi: 10.1093/cid/ciw645 27660236

[24] international-journal-of-pediatric-research-ijpr-9-106.pdf [Internet]. [cited 2024 Jun 3]. Available from: https://web.archive.org/web/20230223084041id_/https://clinmedjournals.org/articles/ijpr/international-journal-of-pediatric-research-ijpr-9-106.pdf?jid=ijpr

[25] Mhlanga TT, Jacobs BKM, Decroo T, Govere E, Bara H, Chonzi P, et al. Virological outcomes and risk factors for non-suppression for routine and repeat viral load testing after enhanced adherence counselling during viral load testing scale-up in Zimbabwe: analytic cross-sectional study using laboratory data from 2014 to 2018. AIDS Res Ther. 2022 Jul 9;19(1):34. doi: 10.1186/s12981-022-00458-z 35810317 PMC9270749

[26] Wasilwa A, Amadi E, Ramadhani HO, Lascko T, Ndaga A, Makokha V, et al. Impact of enhanced adherence counselling on viral re-suppression among adolescents and young persons with persistent viremia. AIDS. 2022 Apr 20; doi: 10.1097/QAD.0000000000003945 38819841 PMC13246298

[27] Masaba RO, Woelk G, Herrera N, Siamba S, Simiyu R, Ochanda B, et al. Standardized enhanced adherence counseling for improved HIV viral suppression among children and adolescents in Homa Bay and Turkana Counties, Kenya. Medicine (Baltimore). 2022 Oct 7;101(40):e30624. doi: 10.1097/MD.0000000000030624 36221325 PMC9542655

[28] Agbornkwai AN, Bita AIG, Mabouna SA, Esa I, Ngongheh AB, Ketum AS, et al. Enhanced Adherence Counselling, Support Groups and Viral Load Suppression amongst HIV-Positive Adolescents in a Tertiary Health Care Facility in Cameroon. Adv Infect Dis. 2022 Oct 20;12(4):685–702.

[29] Bvochora T, Satyanarayana S, Takarinda KC, Bara H, Chonzi P, Komtenza B, et al. Enhanced adherence counselling and viral load suppression in HIV seropositive patients with an initial high viral load in Harare, Zimbabwe: Operational issues. PLOS ONE. 2019 Feb 5;14(2):e0211326. doi: 10.1371/journal.pone.0211326 30721229 PMC6363281

[30] Diress G, Dagne S, Alemnew B, Adane S, Addisu A. Viral Load Suppression after Enhanced Adherence Counseling and Its Predictors among High Viral Load HIV Seropositive People in North Wollo Zone Public Hospitals, Northeast Ethiopia, 2019: Retrospective Cohort Study. AIDS Res Treat. 2020 Apr 21;2020:e8909232. doi: 10.1155/2020/8909232 32373359 PMC7191360

[31] Jobanputra K, Parker LA, Azih C, Okello V, Maphalala G, Kershberger B, et al. Factors Associated with Virological Failure and Suppression after Enhanced Adherence Counselling, in Children, Adolescents and Adults on Antiretroviral Therapy for HIV in Swaziland. PLOS ONE. 2015 Feb 19;10(2):e0116144. doi: 10.1371/journal.pone.0116144 25695494 PMC4335028

