# Supplementary figures and images for: HIV viral load suppression rates among adults and children living with HIV in the North West Region of Cameroon: A call for action!
Source: PLoS One. 2025 Jan 31;20(1):e0316399. doi: 10.1371/journal.pone.0316399 (PMC11785274; doi:10.1371/journal.pone.0316399)

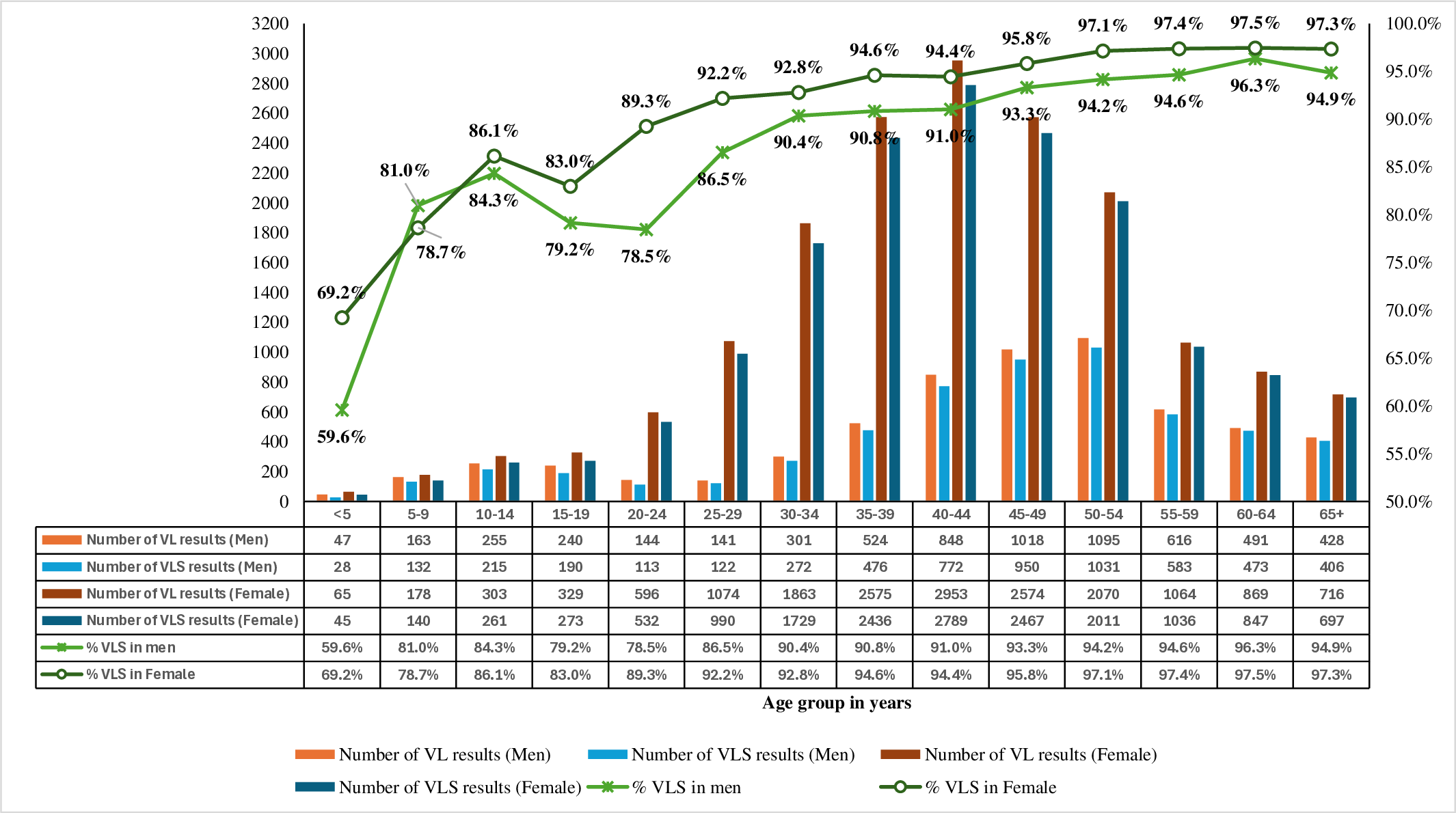

Supplement: S1 Fig — HIV viral load results of PLHIV on ART by sex and age, North West, Cameroon, 2022 (details). (TIF) [file pone.0316399.s001.tif]
